# The PTIP-Associated Histone Methyltransferase Complex Prevents Stress-Induced Maladaptive Cardiac Remodeling

**DOI:** 10.1371/journal.pone.0127839

**Published:** 2015-05-22

**Authors:** Adam B. Stein, Sascha N. Goonewardena, Thomas A. Jones, Parker J. Prusick, Ahmad A. Bazzi, Jane M. Belyavskaya, Makayla M. McCoskey, Rachel A. Dandar

**Affiliations:** 1 Department of Internal Medicine, University of Michigan, Ann Arbor, MI, 48109, United States of America; 2 Central Michigan University College of Medicine, Mt. Pleasant, MI, 48859, United States of America; 3 Department of Biology, Kalamazoo College, Kalamazoo, MI, 49006, United States of America; Rutgers New Jersey Medical School, UNITED STATES

## Abstract

Pressure overload induces stress-induced signaling pathways and a coordinated transcriptional response that begets concentric cardiac hypertrophy. Although concentric hypertrophy initially attenuates wall stress and maintains cardiac function, continued stress can result in maladaptive cardiac remodeling. Cardiac remodeling is orchestrated by transcription factors that act within the context of an epigenetic landscape. Since the epigenetic landscape serves as a molecular link between environmental factors (stress) and cellular phenotype (disease), defining the role of the epigenome in the development and progression of cardiac remodeling could lead to new therapeutic approaches. In this study, we hypothesized that the epigenetic landscape is important in the development of cardiac hypertrophy and the progression to maladaptive remodeling. To demonstrate the importance of the epigenome in HF, we targeted the PTIP-associated histone methyltransferase complex in adult cardiac myocytes. This complex imparts histone H3 lysine 4 (H3K4) methylation marks at actively expressed genes. We subjected PTIP null (PTIP-) mice to 2 weeks of transverse aortic constriction, a stress that induces concentric hypertrophy in control mice (PTIP+). PTIP- mice have a maladaptive response to 2wk of transverse aortic constriction (TAC)-induced pressure overload characterized by cardiac dilatation, decreased LV function, cardiac fibrosis, and increased cell death. PTIP deletion resulted in altered stress-induced gene expression profiles including blunted expression of ADRA1A, ADRA1B, JUN, ATP2A2, ATP1A2, SCN4B, and CACNA1G. These results suggest that H3K4 methylation patterns and the complexes that regulate them, specifically the PTIP-associated HMT, are necessary for the adaptive response to TAC.

## Introduction

Heart failure (HF) is a major cause of morbidity and mortality in the world [1]. In response to hemodynamic and neurohormonal stress, the heart undergoes pathologic remodeling characterized by increased cardiomyocyte volume, interstitial fibrosis, and inflammation, ultimately resulting in cardiac dysfunction and HF [2]. One of the cardinal signs of pathologic cardiac remodeling is cardiac hypertrophy. Although cardiac hypertrophy may initially be a beneficial response to stress, sustained activation of this response can result in pathologic remodeling with cardiac dilatation and decreased cardiac function accompanied by cell death and fibrosis [3, 4]. Clinically, reduced LV function and chamber dilation are associated with increased morbidity and mortality [5–7]. Since HF is a growing epidemic, understanding the molecular mechanisms that regulate the cardiac adaptation to stress and the development of HF are critical to defining new therapeutic avenues.

Gene expression profiles are regulated by transcription factors that act within the context of an epigenetic template that partitions the DNA into actively expressed and repressed regions [8]. The diverse cellular phenotypes observed in multicellular organisms (flies, rodents, and humans) are largely defined by a cell’s epigenetic landscape that is established during development. Early nuclear transfer experiments demonstrated the relative stability of the epigenome in differentiated cells [9]. However, studies in human twins have revealed that environmental factors can induce changes in the epigenome [10]. Studies have demonstrated that an episode of transient hyperglycemia in diabetes can induce epigenetic changes that result in lasting changes in gene expression, i.e. “metabolic memory” [11]. It is postulated that an accumulation of epigenetic changes in the epigenome may contribute to the development of disease [12]. Since HF prevalence increase with aging and is often preceded by risk factors (environmental stressors) and cardiac myocytes are largely terminally differentiated, it stands to reason that epigenetic mechanisms may play a role in the development of HF.

Histone tail methylation marks are one type of epigenetic mark that regulate chromatin structure and the accessibility of transcription factors and transcriptional complexes to enhancer and promoter regions of DNA [13]. Previous work demonstrated rodent and human cardiac hypertrophy and failure are associated with changes in cardiac histone methylation profiles [14, 15]. Zhang et al. further implicated histone methylation profiles in the development of cardiac hypertrophy by deleting a histone de-methylase that removes repressive histone methylation marks, Jmjd2A, in murine cardiac myocytes [16].

One important histone tail methylation mark is tri- methylation of the lysine 4 residue of histone H3 (H3K4me3). H3K4me3 methylation is associated with transcriptionally active chromatin [13]. High levels of H3K4me3 are associated with the 5’ promoter region of nearly all active genes, and there is a strong correlation among this modification, polymerase II occupancy, transcription rates, and histone acetylation [13]. Histone methyltransferase (HMT) complexes impart H3K4me marks. One HMT complex that imparts H3K4me3 marks at the promoter region of actively expressed genes is the PTIP-associated HMT complex [17, 18]. This complex consists of PTIP and the cofactors Ash2L, RbBP5, WDR5, UTX and KMT2B/C, the SET domain containing enzymes that impart tail methylation marks [18]. Previous work has demonstrated that PTIP functions as a link between transcription factors and the rest of the HMT complex [18, 19]. Specifically, PTIP deletion inhibits the ability of the PTIP-associated HMT complex to localize to specific regions of DNA and impart H3K4me3 marks that maintain gene expression [18]. We have previously shown that cardiomyocyte-specific ablation of PTIP causes an overall reduction in H3K4me3 marks and an alteration in cardiomyocyte-specific gene transcription under normal physiologic conditions [20]. PTIP deletion results in a prolongation of action potential duration (APD), augmented intracellular calcium transients, enhanced cardiac function, and a propensity for ventricular premature beats when challenged with isoproterenol and caffeine [20]. Despite these changes, PTIP null mice do not demonstrate cellular or cardiac hypertrophy [20].

In this report, we build on our prior findings to explore the consequences of cardiomyocyte-specific ablation of PTIP in response to cardiac pressure overload induced by 2 weeks of TAC, a hemodynamic stress that induces concentric cardiac hypertrophy. We found that PTIP deletion results in a maladaptive response to TAC. Two weeks after TAC, PTIP- mice rapidly develop LV chamber dilation, LV systolic dysfunction, and cardiac fibrosis. This maladaptive response in PTIP- was accompanied by increased cell death, enhanced fetal gene expression, and an overall shift in gene expression profiles when compared to mice with PTIP. These results provide the first evidence that the PTIP-associated HMT that regulates H3K4me3 marks is necessary for the development of concentric hypertrophy TAC.

## Results

### PTIP- mice have a maladaptive response to pressure overload

**Fig 1:**
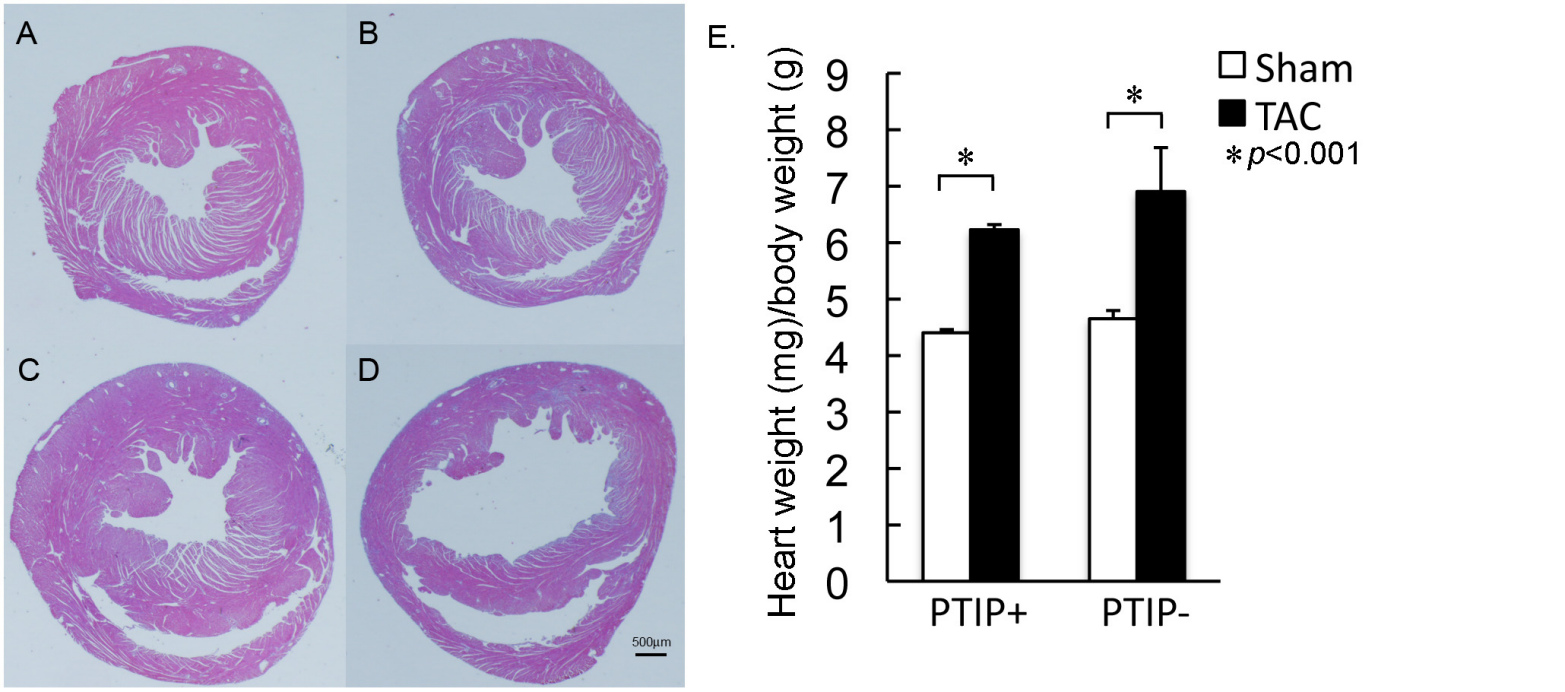
Maladaptive remodeling in PTIP- TAC hearts. PTIP+ and PTIP- hearts were harvested 2wk after sham (**A** and **B**) or TAC (**C** and **D**). H&E staining of ventricular cross-sections in PTIP+ (**A**) and PTIP- (**B**) after sham surgery. After TAC, PTIP+ hearts demonstrate thickened LV walls and preserved chamber size (**C**) in contrast with PTIP- hearts that demonstrate thinner LV walls and a dilated chamber (**D**). After TAC PTIP- (n = 15) and PTIP+ (n = 11) hearts demonstrate a similar increase in heart weight/body weight ratio after TAC (**E**) when compared to PTIP+ sham (n = 8) and PTIP- sham (n = 6). Data shown are means ± SEM.

**Fig 2:**
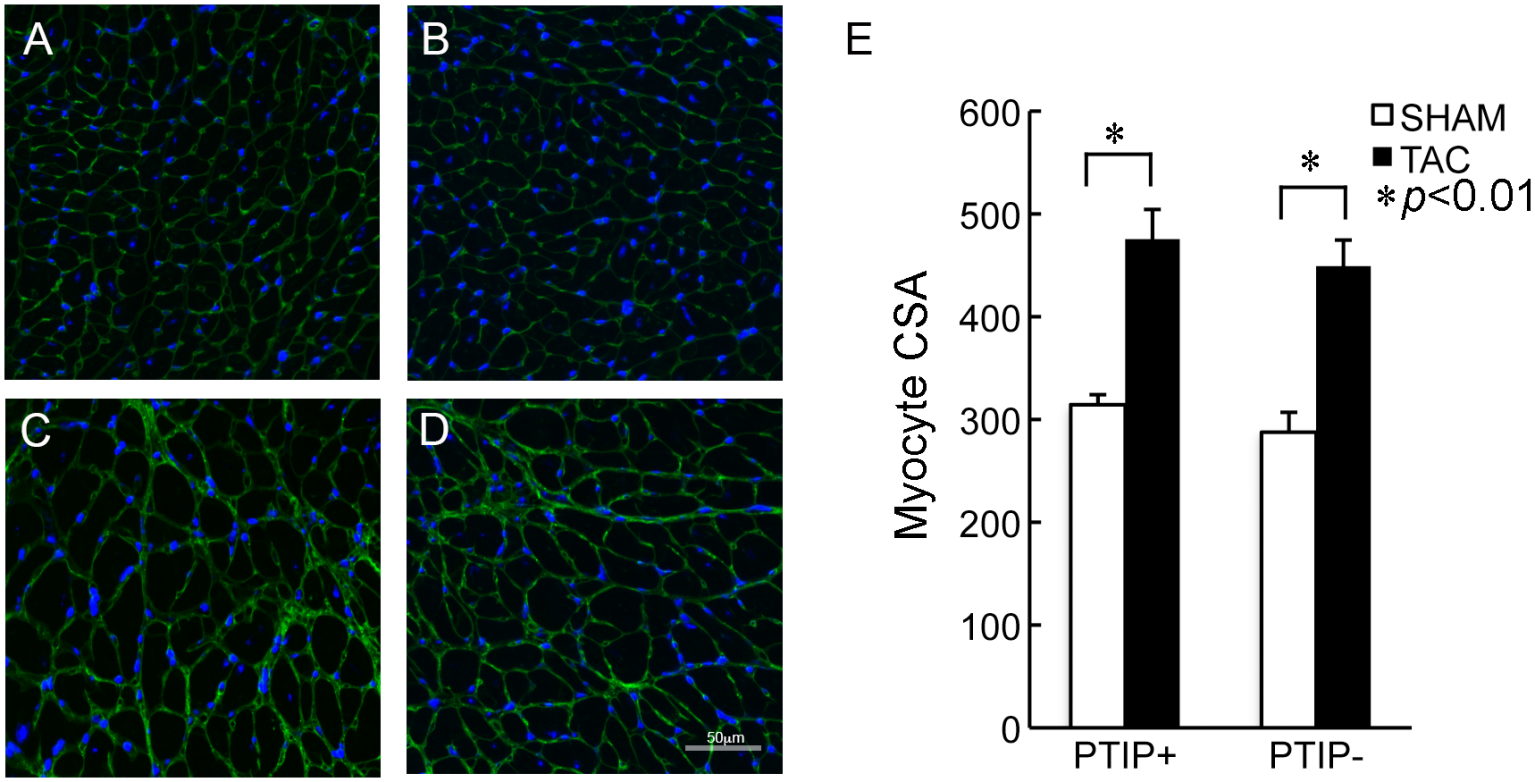
Myocyte cross-sectional area after TAC. FITC-conjugated WGA staining performed on PTIP+ sham (n = 5; panel **A**) and PTIP- sham (n = 5; panel **B**) hearts reveals no significant differences at baseline in myocyte sectional area (CSA). PTIP+ TAC (n = 5; panel **C**) and PTIP- TAC (n = 7; panel **D**) both demonstrate that TAC induces an increase in myocyte CSA. The increase in myocyte CSA in PTIP- hearts is similar to that observed in PTIP+ hearts subjected to TAC (panel **E**). Magnification 400x. At least 200 myocytes from the left ventricle were measured. Data shown are means ± SEM.

The generation of male cardiac-specific PTIP null (PTIP-) and PTIP control (PTIP+) mice has been previously described [20]. Briefly, PTIP+ mice have one allele of a cardiac-specific tamoxifen inducible Cre driver, αMHC-MerCreMer, and two wild type PTIP alleles [20, 21]. PTIP- mice have one allele of αMHC-MerCreMer, and two floxed PTIP alleles. All groups in this study were injected at 8 weeks of age with 20mg/kg of tamoxifen i.p. for 5 days. TAC or sham was performed at 12 weeks of age. To test whether PTIP mediates the ability of the heart to respond to stress, PTIP+ and PTIP- mice were subjected to 2wk of TAC (PTIP+ TAC and PTIP- TAC) or sham surgery (PTIP+ sham and PTIP- sham). H&E staining and heart weight (HW)/body weight (BW) ratio revealed no significant differences in PTIP+ sham and PTIP- sham mice (Fig 1, panel A, B, E). Two weeks after TAC, HW/BW ratio (panel E) ratios increased similarly in both PTIP+TAC and PTIP-TAC hearts compared to PTIP+ sham and PTIP- sham. There were no significant differences in tibia length or body weight between groups (S1 Table). H&E staining revealed that PTIP+ TAC mice developed an increase in LV wall thickness without a gross change in LV chamber diameter (Fig 1, panel C). In contrast, PTIP- TAC mice have thinner LV chamber walls and an increase in LV chamber diameter (Fig 1, panel D). In order to investigate the impact of TAC on myocyte hypertrophy, we performed wheat germ agglutinin staining to assess myocyte cross-sectional area (CSA). We observed no significant differences in myocyte CSA in sham operated PTIP+ and PTIP- hearts (Fig 2, panel A and B). In PTIP+ TAC hearts, there was a significant increase in myocyte CSA (Fig 2, panel C) when compared to PTIP+ sham hearts (Fig 2, panel E). After 2 weeks TAC, PTIP- hearts demonstrated a significant increase in myocyte CSA when compared to PTIP- sham hearts (Fig 2, panel D). The increase in myocyte CSA in PTIP- TAC hearts was similar to the myocyte CSA observed in PTIP+ TAC hearts (Fig 2 panel E).

**Fig 3:**
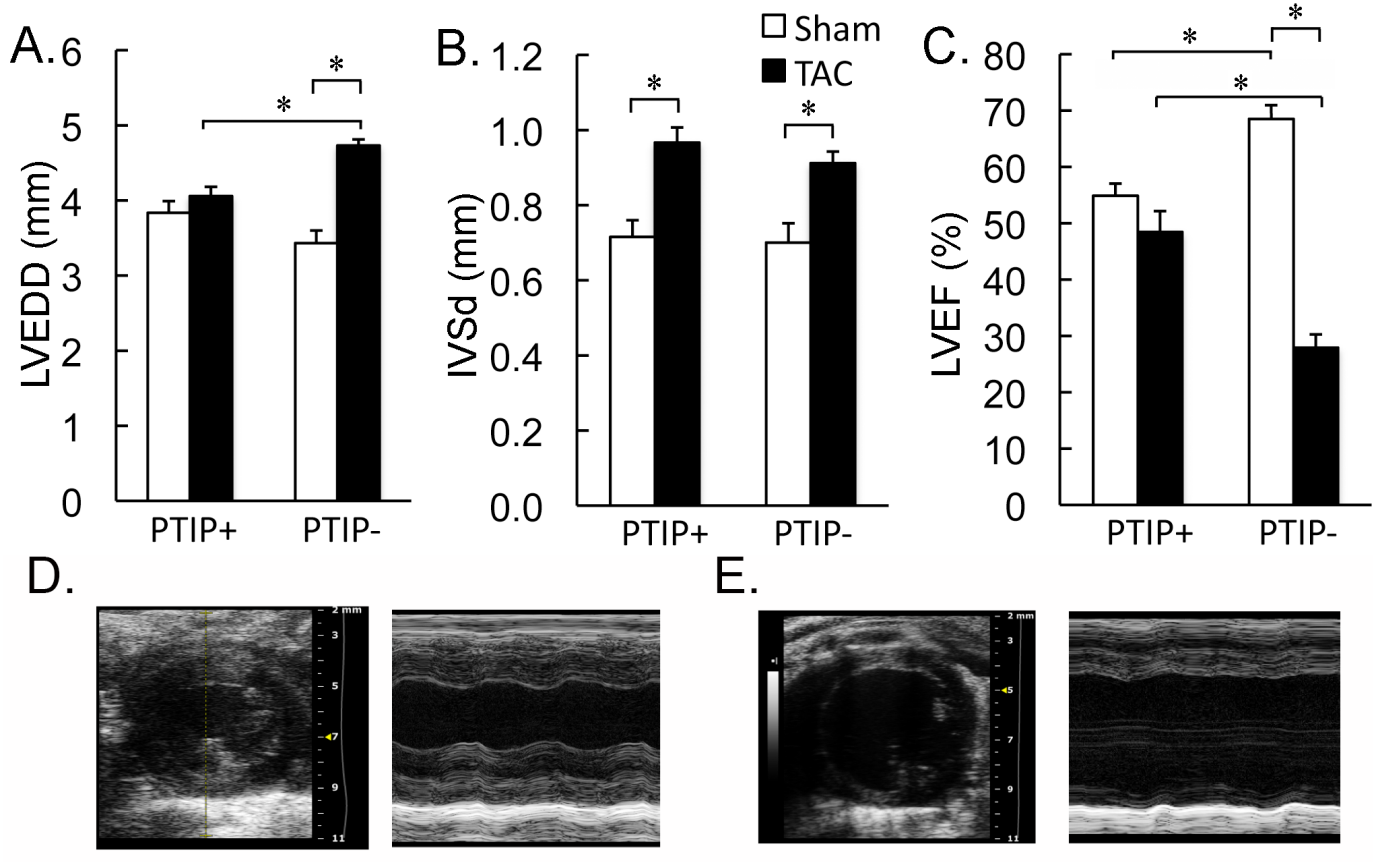
LV chamber dilation and depressed cardiac function in PTIP- TAC hearts. Echo performed on PTIP+ hearts after sham (n = 9) and TAC (n = 10) revealed a significant increase in anterior wall thickness (IVSd; panel B) after TAC without any significant change in LV end diastolic diameter (LVEDD; panel A) or LV ejection fraction (LVEF; panel C). PTIP- hearts after TAC (n = 12) also demonstrated an increase in anterior wall thickness compared with PTIP- sham mice (n = 10). However, PTIP- TAC hearts reveal a significant increase in LVEDD (panel A) and a significant decrease in LVEF when compared to PTIP- sham and PTIP+ TAC hearts. Representative 2D and M-mode images are shown for PTIP+ TAC (panel D) and PTIP- TAC (panel E). Data shown are means ± SEM.

Echo was performed to noninvasively assess the impact of TAC on cardiac structure and function. In sham operated PTIP+ sham and PTIP- sham mice, there were no differences in LV chamber dimensions including LVEDD (LV end-diastolic diameter) and IVSd (anterior wall thickness during diastole) (Fig 3A and 3B). PTIP- sham mice have hyper-contractile cardiac function when compared to PTIP+ sham mice, as previously described [20]. PTIP+ TAC mice demonstrate preserved LVEDD, increased IVSd, and preserved LVEF when compared to PTIP+ sham mice. In contrast, PTIP- TAC mice develop significant LV chamber dilation (LVEDD) and a marked decrease in LVEF and fractional shortening when compared to PTIP- sham and PTIP+ TAC hearts (Fig 3 and S1 Table). Together these results reveal that PTIP is necessary for the development of concentric hypertrophy and the preservation of cardiac function in response to pressure overload. In the absence of PTIP, hearts rapidly progress to failure as revealed by the rapid development of LV chamber dilation and the marked drop in LVEF 2wk post TAC.

### Cardiac fibrosis and cell death in the PTIP- hearts subjected to TAC

**Fig 4:**
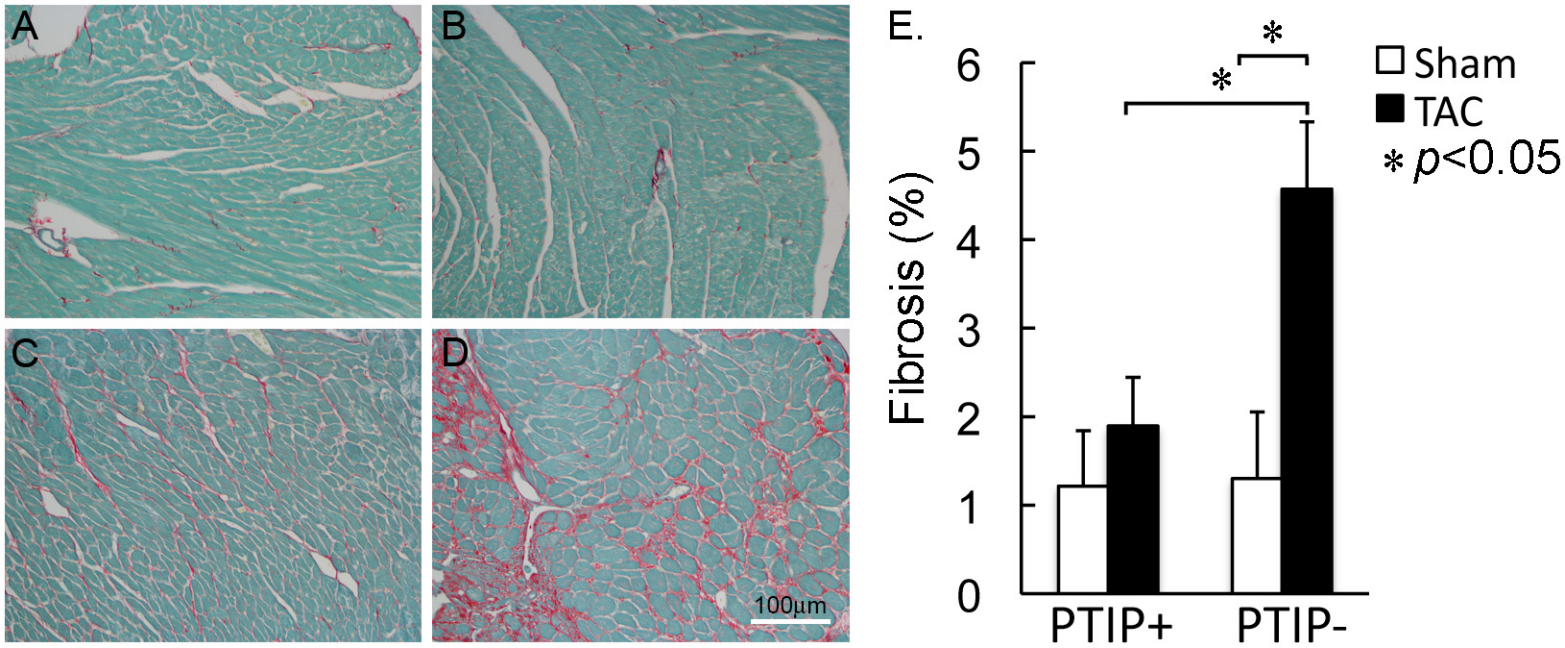
Increased cardiac fibrosis in PTIP- TAC hearts. Fast green and Sirius red staining for collagen revealed no significant differences in sham operated PTIP+ (panel **A**) and PTIP- (panel **B**) hearts. PTIP+ TAC hearts (panel **C**) have an insignificant increase in cardiac fibrosis when compared to PTIP+ sham hearts. PTIP- TAC hearts (panel **D**) have a significant increase in cardiac fibrosis when compared with PTIP-sham and PTIP+ TAC hearts (panel **E**). Data shown are means ± SEM.

Cardiac fibrosis and cell death are hallmarks of heart failure [22, 23]. Picrosirius red staining was performed to assess the impact of PTIP deletion on the TAC-induced development of cardiac fibrosis. As shown in Fig 4, panels A and B, picric acid staining revealed no significant differences in percent fibrosis in PTIP+ sham (1.16±0.65; n = 8) and PTIP- sham (1.19±0.29; n = 6) hearts. In PTIP+ TAC mice (1.86±0.44; n = 11), we observed a small statistically insignificant increase in fibrosis compared to PTIP+ sham mice (panel C). In comparison, PTIP- TAC hearts (4.5±0.71; n = 15) demonstrated a significant increase in cardiac fibrosis (Fig 4, panel D) as compared to PTIP-sham and PTIP+ TAC hearts.

**Fig 5:**
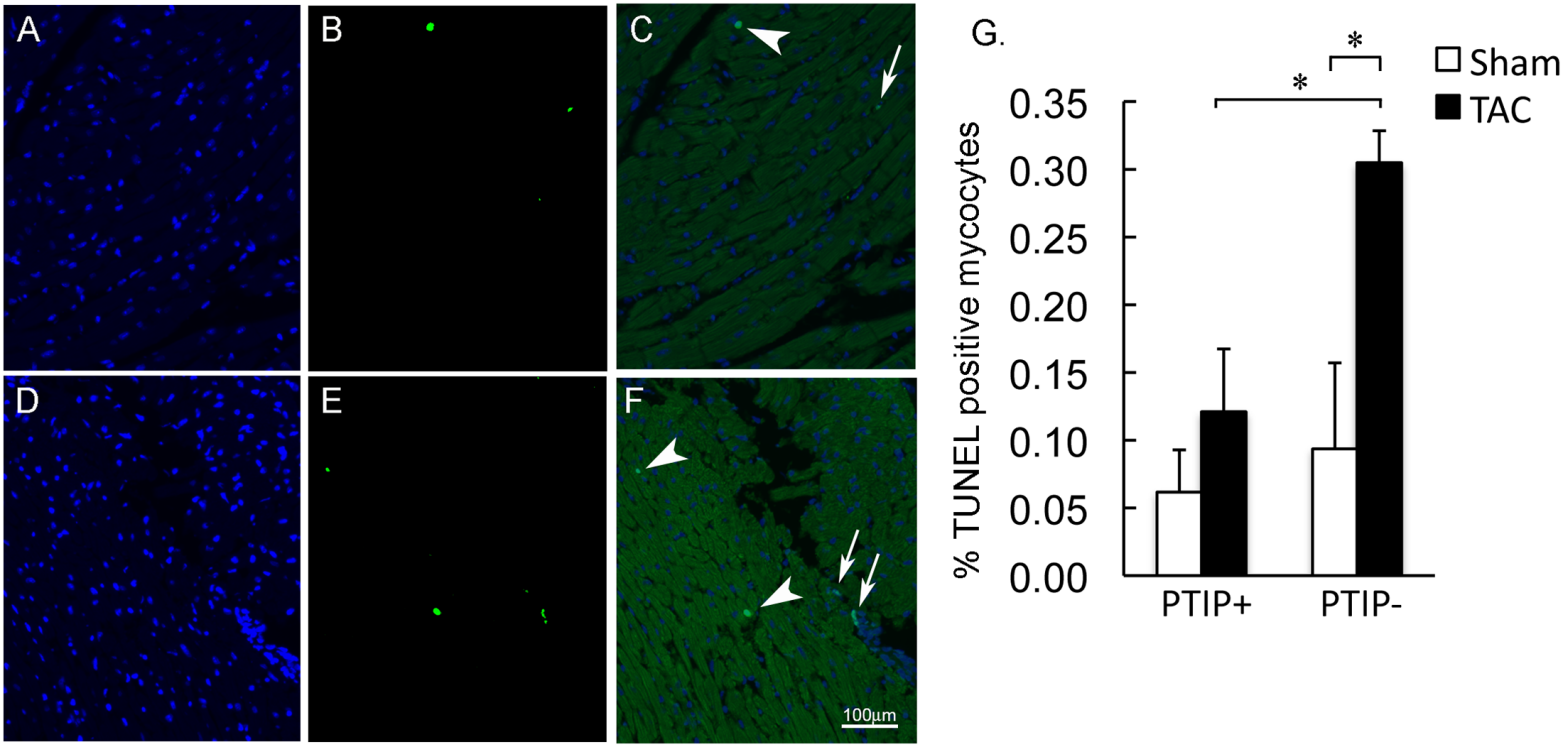
Increase in cell death after TAC in PTIP- hearts. Left ventricles were stained for DAPI and TUNEL. DAPI stain for nuclei is shown in blue for PTIP+ TAC (panel A) and PTIP- TAC (panel D). TUNEL positive nuclei are stained in green for PTIP+ TAC (panel B) and PTIP- TAC (panel E). Overlay of the 2 figures shows TUNEL positive cardiomyocyte nuclei (arrowheads) in PTIP+ TAC hearts (panel C) and PTIP- hearts as well as TUNEL positive non-cardiomyocyte nuclei (arrows). TUNEL staining was quantified by counting 4000 nuclei per heart (panel G). Data shown are means ± SEM.

To assess the impact of PTIP deletion on stress-induced cell death, we performed TUNEL staining on PTIP+ TAC and PTIP- TAC hearts. As shown in Fig 5, TUNEL staining revealed no significant differences in PTIP+ sham (n = 4) and PTIP- sham (n = 4) mice. PTIP+ TAC (Fig 5, panel A-C; n = 7) mice have an insignificant increase in TUNEL stained cardiac myocyte nuclei compared to PTIP+ TAC mice. In contrast, TUNEL in PTIP- TAC hearts (Fig 5, panel D-F; n = 7) revealed a significant increase in TUNEL stained nuclei when compared to PTIP-sham and PTIP+ TAC hearts (Fig 5, panel G).

### Altered gene expression profiles in PTIP- hearts subjected to TAC

**Fig 6:**
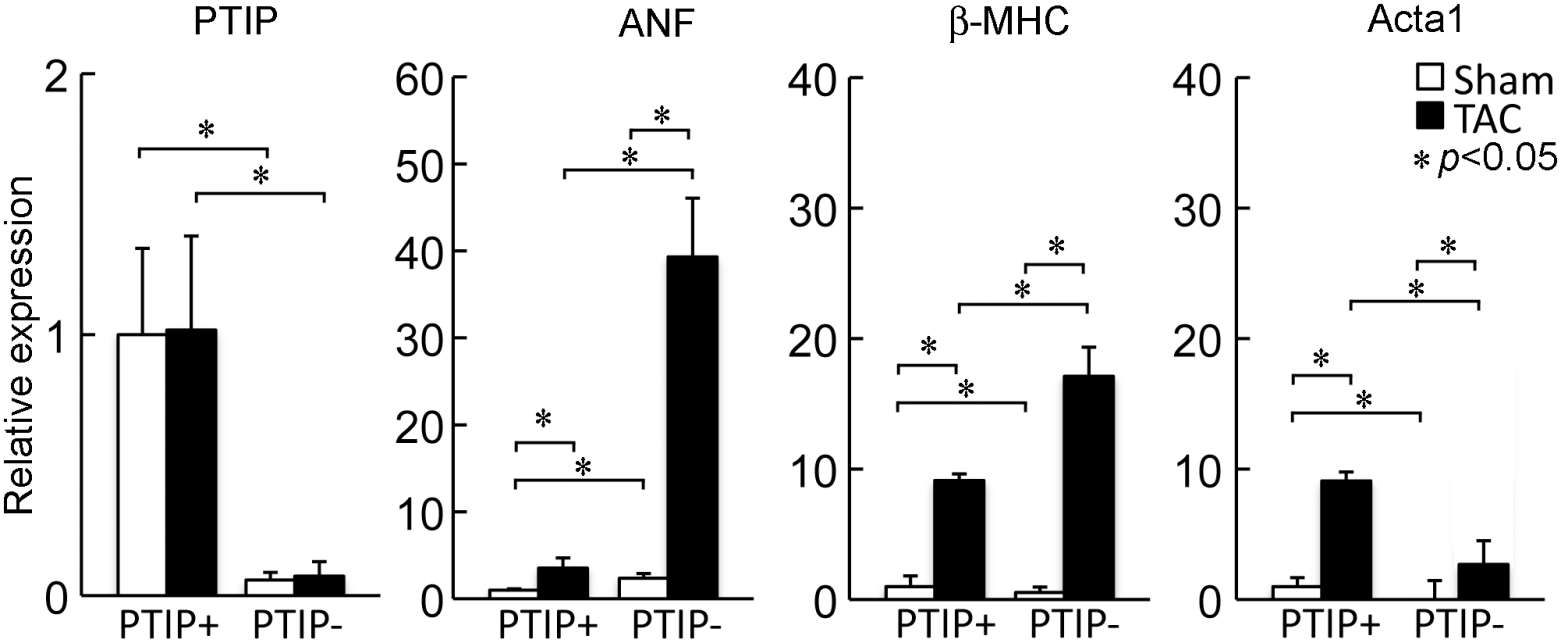
Expression of PTIP and fetal genes after TAC. PTIP expression was significantly attenuated in PTIP- hearts both with and without TAC. Expression of fetal genes after 2wk TAC revealed a significant increase in ANF and β-MHC and a blunted expression of ACTA1 2wk after TAC in PTIP- hearts as compared to PTIP+ TAC hearts. Data shown are means ± SD. Data shown are means ± SD and normalized to GAPDH.

To confirm that PTIP is deleted in PTIP- mice and to determine whether PTIP levels change in response to TAC, we performed qPCR on TAC and sham animals (n = 4-6/group). As shown in Fig 6, PTIP mRNA levels are significantly attenuated in PTIP- sham and PTIP- TAC animals and PTIP mRNA levels do not change significantly after TAC (PTIP+ sham vs. PTIP+ TAC).

LV remodeling and the development of hypertrophy and heart failure is accompanied by the induction of a fetal gene expression program. Accordingly, we measured the fetal genes ANF, β-MHC and Acta1 by qPCR in PTIP+ sham, PTIP- sham, PTIP+ TAC and PTIP- TAC (Fig 5, panels B, C, and D). At baseline, ANF levels were elevated in PTIP- sham animals as compared to PTIP+ sham animals. After TAC, ANF increased in PTIP+ TAC as compared to PTIP+ sham. We observed a robust increase in ANF levels in PTIP-TAC hearts as compared to PTIP- sham and PTIP+ TAC. At baseline, β-MHC and Acta1 expression was lower in sham PTIP- animals than in PTIP+ animals. After TAC, β-MHC expression increased dramatically in PTIP- TAC hearts as compared to PTIP+ TAC hearts. Acta1 expression increased in both PTIP+ and PTIP- subjected to TAC, however the increase in PTIP- TAC animals was significantly less than that observed in PTIP+ TAC animals. Together these results reveal that the TAC-induced maladaptive remodeling in PTIP- results in an abnormal expression of fetal genes.

Since the PTIP-associated HMT complex regulates transcription via H3K4me3 marks, we rationalized that TAC would result in abnormal gene expression profiles in PTIP- mice. Accordingly, gene expression array was performed on PTIP+ TAC (n = 5) and PTIP- TAC (n = 4) at 2wk post TAC (S1 Fig). Gene expression array revealed that a total of 386 genes that were differentially expressed in PTIP+ TAC and PTIP- TAC. Of these genes, 230 were decreased and 156 were increased in PTIP- TAC hearts compared to PTIP+ TAC hearts. The abundance of down-regulated genes in PTIP- TAC hearts supports the concept that PTIP regulates actively expressed genes.

**Fig 7:**
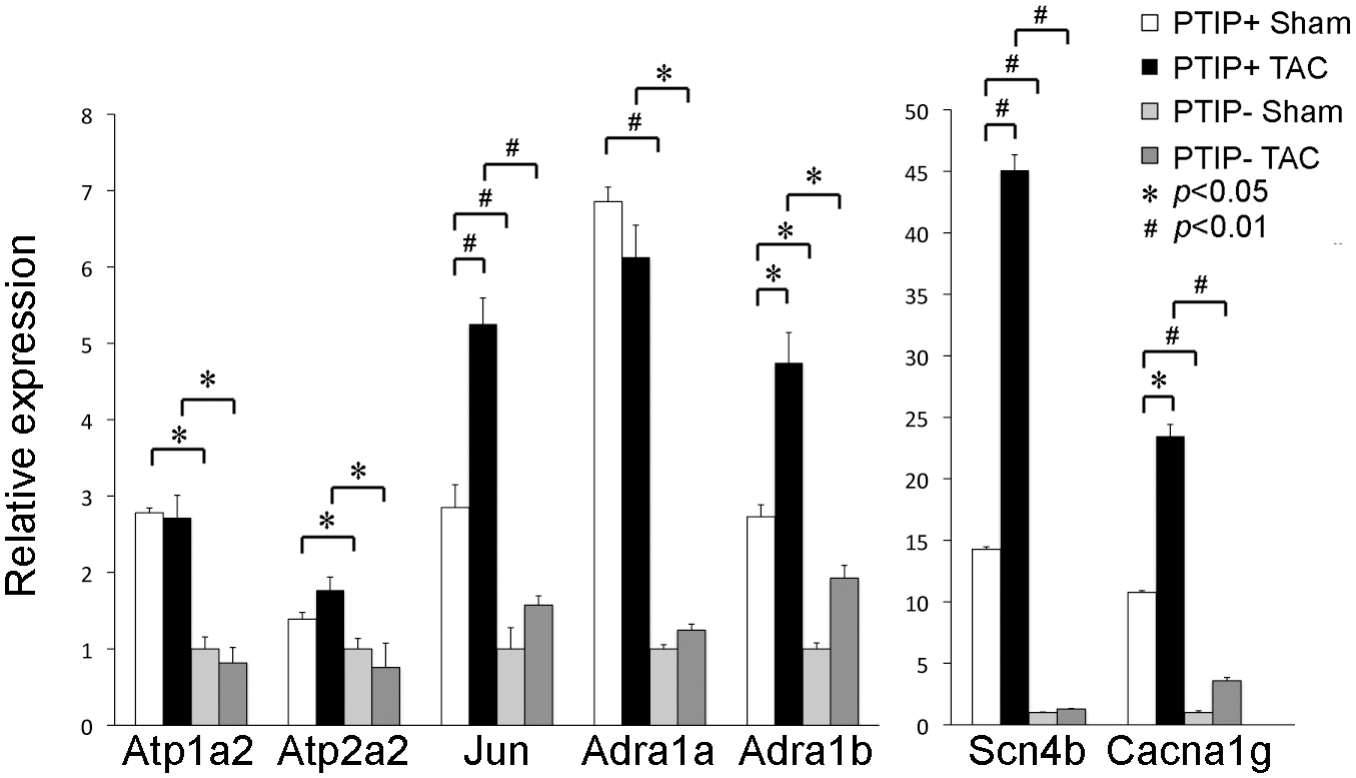
Attenuated expression of cardiac genes in PTIP- mice after sham or TAC. qPCR was performed to define the expression of gene expression array-identified genes in PTIP- hearts. These studies were performed 3d after TAC. PTIP deletion resulted in attenuated expression of the calcium handling genes ATP1A2, ATP2A2, JUN, the alpha-adrenergic receptors ADRA1A and ADRA1B, SCN4B, and CACNA1G after TAC. Data shown are means ± SD and normalized to GAPDH.

In order to confirm our gene expression array data and identify genes that could contribute to the maladaptive stress response observed in PTIP- TAC hearts, we performed qPCR for specific genes that were identified by gene expression array. Since our phenotype was already evident after only 2wk TAC, we rationalized that gene expression profiles would be abnormal early after TAC. Thus, qPCR was performed on PTIP+ TAC and PTIP- TAC hearts 3d after TAC. Gene array revealed that ATP1A2 was attenuated in PTIP- TAC hearts. ATP1A2 is a Na(+), K(+)- ATPase that regulates murine cardiac contraction. qPCR revealed a significant attenuation of ATP1A2 in PTIP- hearts compared to PTIP+ hearts (Fig 7). ATP2A2 is a major sarcoplasmic/endoplasmic reticulum Ca(2+)-ATPase that regulates intracellular calcium levels and contractility. Reduced ATP2A2 expression is associated with cardiac failure, and normalizing ATP2A2 expression is being pursued as a therapeutic target in heart failure [24–26]. As shown in Fig 7, ATP2A2 levels are reduced in PTIP- TAC and PTIP- sham hearts as compared to PTIP+ TAC and PTIP+ sham. JUN is an AP-1 transcription factor that protects the heart from maladaptive cardiac remodeling [27]. qPCR for JUN expression revealed that the TAC-induced increase in JUN expression in PTIP+ mice is significantly blunted in PTIP- mice.

α1-adrenergic receptors play a critical role in cardiac biology and are necessary for the adaptive response to TAC [28]. Accordingly, we performed qPCR for ADRA1A and ADRA1B. As shown in Fig 7, ADRA1A and ADRA1B demonstrated a significant attenuation in PTIP- sham hearts compared to PTIP+ sham hearts and in PTIP-TAC hearts compared to PTIP+ TAC hearts. SCN4B forms a component of the sodium channel and has been associated with congenital long QT syndrome [29]. Gene expression array revealed SCN4B was strongly downregulated in our gene expression array in the absence of PTIP. qPCR for SCN4B expression confirmed SCN4B expression was attenuated in PTIP- Sham and PTIP- TAC hearts when compared to PTIP+ Sham and PTIP+ TAC hearts. CACNA1G encodes for an alpha subunit that regulates the T-type calcium current. CACNA1G deletion results in increased fibrosis and decreased LV function after TAC [30]. qPCR for CACNA1G demonstrated an increase in CACNA1G expression after TAC in PTIP+ hearts. Cacan1g expression was significantly attenuated in PTIP- sham and TAC hearts (Fig 7). Taken together, these results reveal that the absence of PTIP results in an inability to maintain and upregulate genes that are known to be important in cardiac homeostasis and the ability of the heart to adapt to stress.

### Attenuation of H3K4me3 marks at specific genes in PTIP- hearts

**Fig 8:**
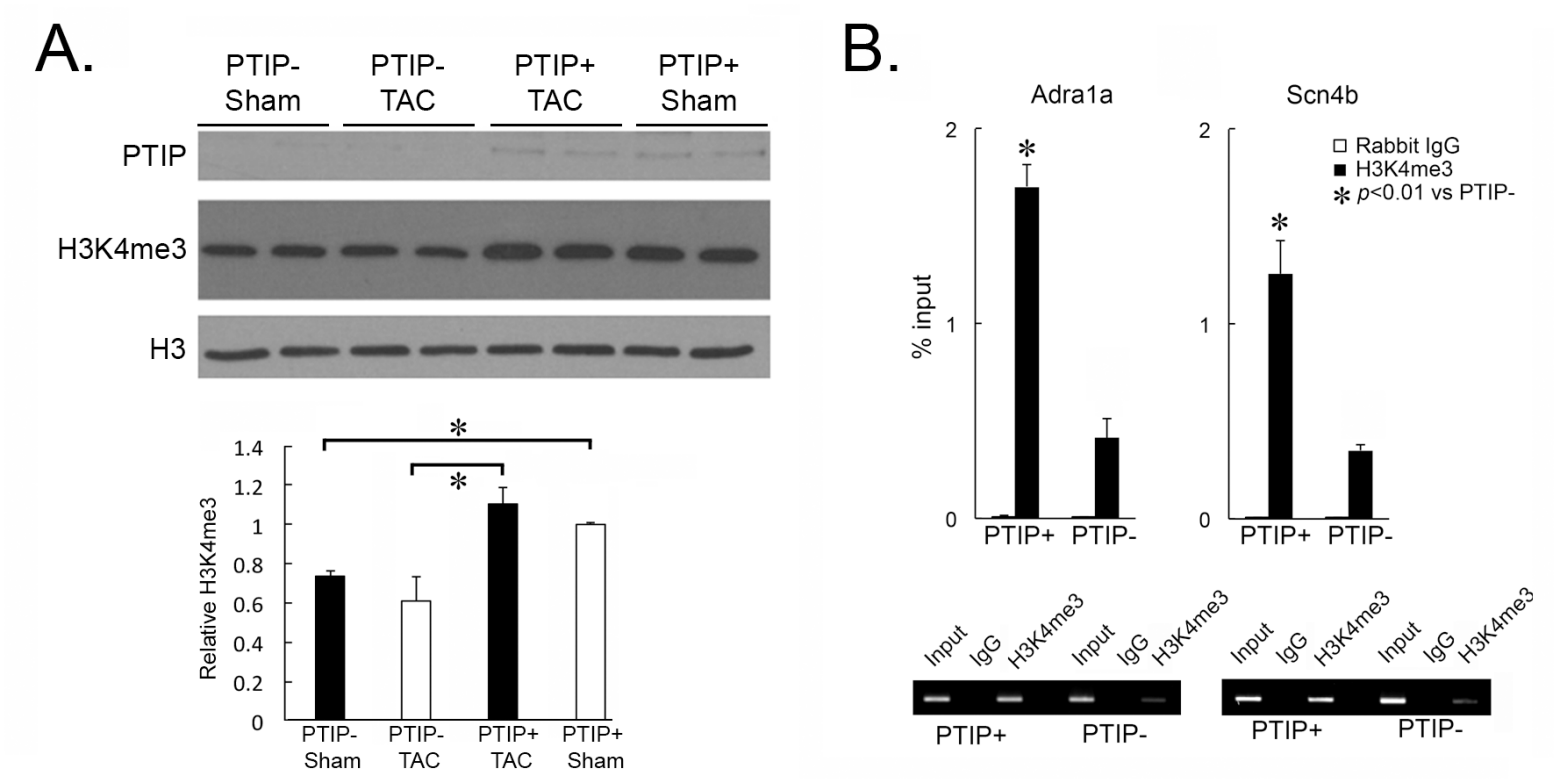
PTIP deletion attenuates H3K4me3 marks. Immunoblot analysis for PTIP and H3K4me3 was performed to determine the impact of PTIP deletion and TAC on global H3K4me3 levels and PTIP expression. As shown in panel A, PTIP deletion results in a significant attenuation in global H3K4me3 marks when normalized to total histone H3 levels. TAC had no significant impact on PTIP levels or H3K4me3 levels. To determine whether PTIP regulates H3K4me3 levels at the promoter region of specific genes, ChIP-qPCR was performed to assess H3K4me3 enrichment at the promoter region of ADRA1A and SCN4B in PTIP- and PTIP+ mice (panel B). ChIP was performed with an anti-H3K4me3 antibody (black) and a non-specific Rabbit IgG antibody (white). Data reveal a significant decrease enrichment of H3K4me3 marks in PTIP- hearts (n = 6) as compared to PTIP+ hearts (n = 6) at the Adra1a and SCN4b promoter. The amplified ChIP-qPCR products were run on a gel with a 2% input (panel B, below). Data shown are means ± SEM.

The PTIP-associated HMT regulates H3K4me3 marks at actively expressed genes. We have previously demonstrated that PTIP deletion results in an attenuation of global cardiac H3K4me3 marks. In order to determine the impact of TAC and PTIP deletion on global H3K4me3 marks we performed western blot analysis for PTIP and H3K4me3 in PTIP+ and PTIP- hearts after sham or TAC surgery. As demonstrated in Fig 8, global H3K4me3 marks are attenuated in PTIP- hearts compared to PTIP+ hearts. This reduction is evident in sham and persists after TAC. We observed no changes in global H3K4me3 levels or PTIP expression in PTIP+ sham and TAC hearts suggesting that 2wk TAC is not associated with changes in global H3K4me3 levels. To confirm these global finding and demonstrate that PTIP-associated H3K4me3 marks are associated with promoter regions, we performed gene-specific ChIP-qPCR for ADRA1A and SCN4B using an anti-H3K4me3 antibody and a control rabbit IgG antibody in PTIP+ sham and PTIP- sham hearts (n = 3/group). Analysis of ChIP-qPCR at the promoter region of these genes reveals a greater than two fold decrease in H3K4me3 enrichment at the promoter region of ADRA1A and SCN4B (Fig 8).

## Discussion

Our data demonstrate that absence of the PTIP-associated HMT results in a maladaptive response to pressure overload. After just two weeks of TAC, PTIP- mice develop cardiac dilation, a marked decrease in LV systolic function, an increase in cardiac fibrosis, and an increase in cell death. These phenotypic consequences were underscored by an abnormal shift in gene expression profiles and altered H3K4me3 profiles.

Epigenetic marks are acquired during development and partition the DNA into transcriptionally active and repressed regions. H3K4me3, are heritable, stable, and conserved in yeast, flies and humans [8]. Previous work has demonstrated that histone methylation profiles change during the development of hypertrophy and failure in rodents and humans [14, 15]. The objective of this study was to determine whether H3K4me3 marks and the epigenetic machinery that impart them are necessary for the cardiac stress response. In this study, we utilized a PTIP null murine model to demonstrate that the PTIP-associated HMT and the associated H3K4me3 marks regulate the response of adult cardiac myocytes to pressure overload; providing evidence that the epigenome is a key regulator of the stress response.

These results provide proof-of—concept that epigenetic mechanisms may play a role in defining how and why maladaptive cardiac remodeling progresses in patients. The role of the epigenome in the development of disease is widely studied because epigenetic mechanisms provide a molecular explanation for how transient environmental stressors induce persistent changes in gene expression profiles and cellular homeostasis. Data looking at twins reveals that epigenetic profiles can diverge over time due to environmental factors and stochastic events, and transient hyperglycemia results in long lasting changes in gene expression in diabetes via epigenetic mechanisms [10, 11]. Epigenetic changes may be particularly important in cardiac myocytes because this cell type is largely terminally differentiated with a low rate of turnover [31]. As a result, adult myocytes in the elderly, a population with a high incidence of HF, have had ample time to be subjected to environmental stressors (hypertension and diabetes) and stochastic events. To demonstrate that the epigenetic mechanisms mediate heart disease, we must first demonstrate that changing the epigenome can regulate cardiac disease states. Our work suggests that epigenetic mechanisms, as modeled by PTIP deletion, mediate cardiac remodeling and arrhythmogenesis, two major components of cardiac disease, and highlight the potential importance of the epigenome in mediating cardiac biology and disease. Work by others also supports the importance of the epigenome in cardiac homeostasis as well [16, 32].

An interesting finding of this study is that PTIP deletion results in an increase in cardiac myocyte cell death after TAC. In addition to its role in regulating H3K4me2/3 marks PTIP is known to contain 6 BRCT domains and is known to be important in DNA damage and repair pathways. After DNA damage, PTIP localizes to damaged nuclear foci that contain gH2AX, 53BP1 (p53-binding protein 1) and the MRN complex to repair DNA damage [33–36]. Cell death is an important regulator of cardiovascular disease states including myocardial infarction and heart failure [37, 38]. Although our data demonstrate increased cell death rates in PTIP- TAC hearts, it remains unclear whether the myocyte cell death is a result of aberrant expression of genes that regulate cell death or due to a direct role for PTIP in DNA break repair. The exact role that PTIP plays in mediating cardiac myocyte cell death warrants further investigation.

This study reveals that the PTIP-associated HMT is a key regulator of H3K4me3 marks in adult cardiac myocytes. Our western blot data (Fig 8) reveals that PTIP deletion decreases global H3K4me3, and that this detriment persists during TAC. This data also demonstrates that PTIP levels and H3K4me3 marks do not change globally with TAC. Although the relevance of PTIP in the development of cardiac hypertrophy is not readily evident from this data, others have demonstrated that gene-specific H3K4me3 marks do change in human and rodent models of hypertrophy/failure [14, 15]. It is plausible, and likely, given the observed global changes in H3K4me3 marks, that the level of expression of PTIP during stress is not as important as the mechanisms that localize PTIP to specific target genes. Due to experimental limitations, we were not able to implicate PTIP directly in the regulation of potential target genes, and, thus, we are not able to establish a definitive mechanism that explains our phenotypic findings.

To further define the mechanistic role of PTIP in disease states, future studies will need to be performed to determine how PTIP targets certain genes in adult hearts. PTIP was originally discovered based on its interaction with Pax2 [18]. Pax2 is a DNA binding protein important in kidney development. In this model, the specificity of the PTIP:DNA interaction is intrinsic to Pax2 DNA binding specificity [18]. A similar interaction between PTIP and Pax5 has been shown in B-cells [19]. To our knowledge, the Pax-family of proteins do not play a role in the adult heart. Recently, Pitx2 was shown to interact with PTIP, however Pitx2 is not known to play a key role in the biology of adult ventricular tissue [39]. Thus, it remains unknown what DNA binding protein in the adult heart interacts with PTIP.

## Methods

### Animals

Mice were kept according to NIH guidelines. All animal studies were reviewed and approved by the University Committee on Use and Care of Animals at the University of Michigan. PTIP+ and PTIP- mice were created as previously described [20]. All mice used in this study underwent i.p. tamoxifen (20mg/kg) injection at 8 weeks of age and were used for study at 12 weeks of age. We have previously demonstrated that this dose and timing of tamoxifen results in no significant functional consequences [20].

### Surgery

Twelve-week old male PTIP+ and PTIP- mice were subjected to TAC. Mice were anesthetized with isoflurane. The transverse aorta was approached via a suprasternal approach. Once the transverse aorta was identified, a 5–0 silk suture was placed around the aorta between the innominate and left common carotid arteries. The suture was tied tight around the aorta and a 27-gauge needle. The needle was removed, resulting in highly reproducible constriction. Sham animals underwent identical surgical procedures with the exception of the suture placement. Animals underwent echo 2 weeks after TAC or sham to noninvasively assess LV chamber structure and function as previously described [20]. Two weeks after TAC, hearts were harvested and weighed to assess hypertrophy and prepared for various assays.

### Histology and TUNEL assay

2-weeks after surgery, mice were euthanized, hearts quickly removed and fixed in 4% paraformaldehyde overnight for standard histology [hematoxylin/eosin (H&E) stainings]. Fast green/Sirius red staining sections were stained using 0.1% Fast green/0.1% Sirius red for 30 min. Wheat Germ Agglutinin staining slides were incubated with the WGA lectin (Alexa Fluor 488 conjugate, *Life technologies*) for 2 hours. TUNEL staining sections were labeled using the TUNEL Apoptosis Detection Kit (*Millipore*, *Cat*. *#17–141*).

### qPCR and Gene Expression Array

LV samples were harvested, and RNA was isolated and sent to the University of Michigan Comprehensive Cancer Center Affymetrix and Microarray Core Facility or used for qPCR with TaqMan probes. For gene expression array we used Mouse Gene ST2.1 strip arrays processed using the Ambion WT kit. Robust multi-array average was used to normalize and fit log2 expression values using the Affy package of bioconductor [40]. Linear models where used to compare one group to another using the LIMMA package of bioconductor [41]. Array quality weights were used in the model to downweight genes considered less reproducible [42]. P-values were adjusted for multiple comparisons using false discovery rate [43].

### Western blotting

Ventricles were harvested, snap frozen, pulverized, and homogenized. The cytosolic fraction was solubilized in a buffer with 25 mmol/L Tris-HCl [pH 7.4], 0.5 mmol/L EDTA, 0.5 mmol/L EGTA, 1 mmol/L PMSF, 1 mmol/L DTT, 25 μg/mL leupeptin, 25 mmol/L NaF, and 1 mmol/L Na_3_VO_4_. After centrifuging, the resulting pellet containing the nuclei was pelleted through a sucrose gradient. The pellet was solubilized in a buffer containing 25 mmol/L HEPES [pH 7.8], 420 mmol/L NaCl, 1.5 mmol/L MgCl2, 0.2 mmol/L EDTA, 0.5 mmol/L DTT, 25% Glycerol, 0.5 mmol/L APMSF 15ug/ml aprotinin, 5ug/ml leupeptin, and 2ug/ml pepstatin. The resulting supernatants were used as the nuclear fraction. Western immunoblot analysis was performed with standard sodium dodecyl sulfate—polyacrylamide gel electrophoresis immunoblotting techniques. Antibodies utilized were anti-H3 (Abcam, ab1791), anti-H3K4me3 (Abcam, ab8580), and rabbit anti-PTIP. Blots were quantified by densitometry and normalized to H3 levels.

### ChIP-qPCR

Ventricles were harvested and minced into 1- to 2-mm^2^ pieces. For H3K4me3 marks, minced tissue was cross-linked with 1% formaldehyde in culture media for 15 minutes. The protocol was then followed as previously described.[20] Antibodies for ChIP include rabbit IgG (4 μg) and rabbit anti-H3K4me3 (Abcam, ab8580) (4 μg). Primers to amplify the ADRA1A promoter are 5’- CGCTCTTTTCTGTGTCCAGGAAC and 5’-GGCTCGCTTCTTGCTTCTCC and SCN4B 5’ ATTCCATCCCAAGCCCCC and 5’ AAAGCCCAGTGCCCAGCCATCTCGC.

### Statistics

Statistical significance was calculated using 2-tailed Student’s *t* test to compare 2 groups and analysis of variance with Bonferroni correction to compare multiple groups. *P* values of less than 0.05 were considered significant. All data shown are mean ± SD unless otherwise specified.

## Supporting Information

S1 Fig Gene expression array analysis of PTIP+ TAC and PTIP- TAC. PTIP+ TAC (n = 4) and PTIP- TAC (n = 5) hearts were subjected to TAC for 2 weeks. LV tissue was harvested and sent for gene expression array analysis at the University of Michigan DNA sequencing Core. Array data for the individual hearts are presented and labeled PTIP- TAC #1-#5 and PTIP+ TAC #1-#4. Data are organized such that the genes with the most significant p-value are closest to the top. “Log2 fold change” numbers that are positive represent genes that increased in PTIP- TAC hearts and negative “log2 fold change” numbers represent genes that decreased in PTIP- TAC mice.
(XLSX)

S1 Table Additional Echo and Pathology Parameters. Additional echocardiography and pathology parameters are included. Data shown are means ± SD. LVESD is LV chamber diameter in systole. PWd is posterior wall in diastole.* p<0.05 vs. PTIP-Sham; # p<0.05 vs. PTIP+ Sham; $ p< 0.05 vs. PTIP+ TAC.
(DOCX)
