# Supplementary material for: The PTIP-Associated Histone Methyltransferase Complex Prevents Stress-Induced Maladaptive Cardiac Remodeling
Source: PLoS One. 2015 May 22;10(5):e0127839. doi: 10.1371/journal.pone.0127839 (PMC4441468; doi:10.1371/journal.pone.0127839)
Supplement: S1 Table — Additional echocardiography and pathology parameters are included. Data shown are means ± SD. LVESD is LV chamber diameter in systole. PWd is posterior wall in diastole.* p<0.05 vs. PTIP-Sham; # p<0.05 vs. PTIP+ Sham; $ p< 0.05 vs. PTIP+ TAC. (DOCX) [file pone.0127839.s002.docx]

S1 Table

Additional echo and path parameters.

|  | PTIP+ Sham | PTIP+ TAC | PTIP- Sham | PTIP- TAC |
| --- | --- | --- | --- | --- |
| Body weight (g) | 28.8 ± 3.2 | 26.7 ± 3.1 | 28.8 ± 3.2 | 26.7 ± 3.1 |
| Tibia length (mm) | 22.7 ± 0.4 | 22.8 ± 0.7 | 22.7±0.3 | 22.6 ± 0.6 |
| Heart rate (bpm) | 468 ± 38 | 477 ± 42 | 481 ± 55 | 441 ± 37 |
| LVESD (mm) | 2.9 ± 0.4 | 3.0 ± 0.5 | 2.27 ± 0.4# | 4.1 ± 0.4*$ |
| PWd (mm) | 0.65 ± 0.14 | 0.93 ± 0.17# | 0.64 ± 0.20 | 0.82 ± 0.12* |
| Fractional shortening | 0.24 ± 0.03 | 0.25 ± 0.07 | 0.34 ± 0.42# | 0.13 ± 0.04*$ |

Data shown are means ± SD. LVESD is LV chamber diameter in systole. PWd is posterior wall in diastole.* p<0.05 vs. PTIP-Sham; # p<0.05 vs. PTIP+ Sham; $ p< 0.05 vs. PTIP+ TAC
